# Sequential Epidermal Growth Factor Receptor Tyrosine Kinase Inhibitor-Induced Radical Surgery in Oligometastatic Non–Small Cell Lung Cancer: A Case Report

**DOI:** 10.1093/oncolo/oyad339

**Published:** 2024-01-10

**Authors:** Huaiyu Wang, Zaiwen Fan, Xiaoming Yang, Qing Wu, Suxin Jiang, Jingna Zhu, Jie Liu, Chuanhong Zhou, Yinghui Liu, Xia You, Yong Han

**Affiliations:** Thoracic Surgery Department, Air Force Medical Center, PLA, Beijing, People’s Republic of China; Medical Oncology Department, Air Force Medical Center, PLA, Beijing, People’s Republic of China; Anesthesiology Department, Air Force Medical Center, PLA, Beijing, People’s Republic of China; Thoracic Surgery Department, Air Force Medical Center, PLA, Beijing, People’s Republic of China; Thoracic Surgery Department, Air Force Medical Center, PLA, Beijing, People’s Republic of China; Thoracic Surgery Department, Air Force Medical Center, PLA, Beijing, People’s Republic of China; Thoracic Surgery Department, Air Force Medical Center, PLA, Beijing, People’s Republic of China; Thoracic Surgery Department, Air Force Medical Center, PLA, Beijing, People’s Republic of China; Thoracic Surgery Department, Air Force Medical Center, PLA, Beijing, People’s Republic of China; The State Key Lab of Translational Medicine and Innovative Drug Development, Jiangsu Simcere Diagnostics Co., Ltd, Nanjing, Jiangsu Province, People’s Republic of China; Thoracic Surgery Department, Air Force Medical Center, PLA, Beijing, People’s Republic of China

**Keywords:** EGFR L718Q, oligometastatic NSCLC, downstaging, whole course medical management

## Abstract

Sequential regimens in patients with epidermal growth factor receptor (EGFR) mutation-positive non–small cell lung cancer (NSCLC) can overcome tyrosine kinase inhibitor (TKI) resistance and maximize clinical benefit. Patients with advanced NSCLC can achieve excellent tumor control after a period of EGFR-TKI treatment. Patients may benefit from additional local treatment, such as surgery or radiation therapy, once the tumor is under control. Here, we present a case of a patient with advanced oligometastatic NSCLC with EGFR mutations who achieved downstaging through sequential EGFR-TKI-based precision medicine allowing resection of residual disease.

## Teaser

This case report describes a patient with advanced oligometastatic non–small cell lung cancer with EGFR mutations who achieved downstaging through sequential EGFR-TKI-based precision medicine allowing resection of residual disease.

**Key Points**

- We report one case of a patient with oligometastatic non–small cell lung cancer achieving downstaging and surgical outcomes by sequential targeted therapy.
- The acquisition of a new genetic mutation EGFR L718Q led to osimertinib resistance; the patient was then treated with afatinib, with disease control since.
- The overall benefit time of patient was more than 51 months.

## Case Presentation

**Figure 1.**
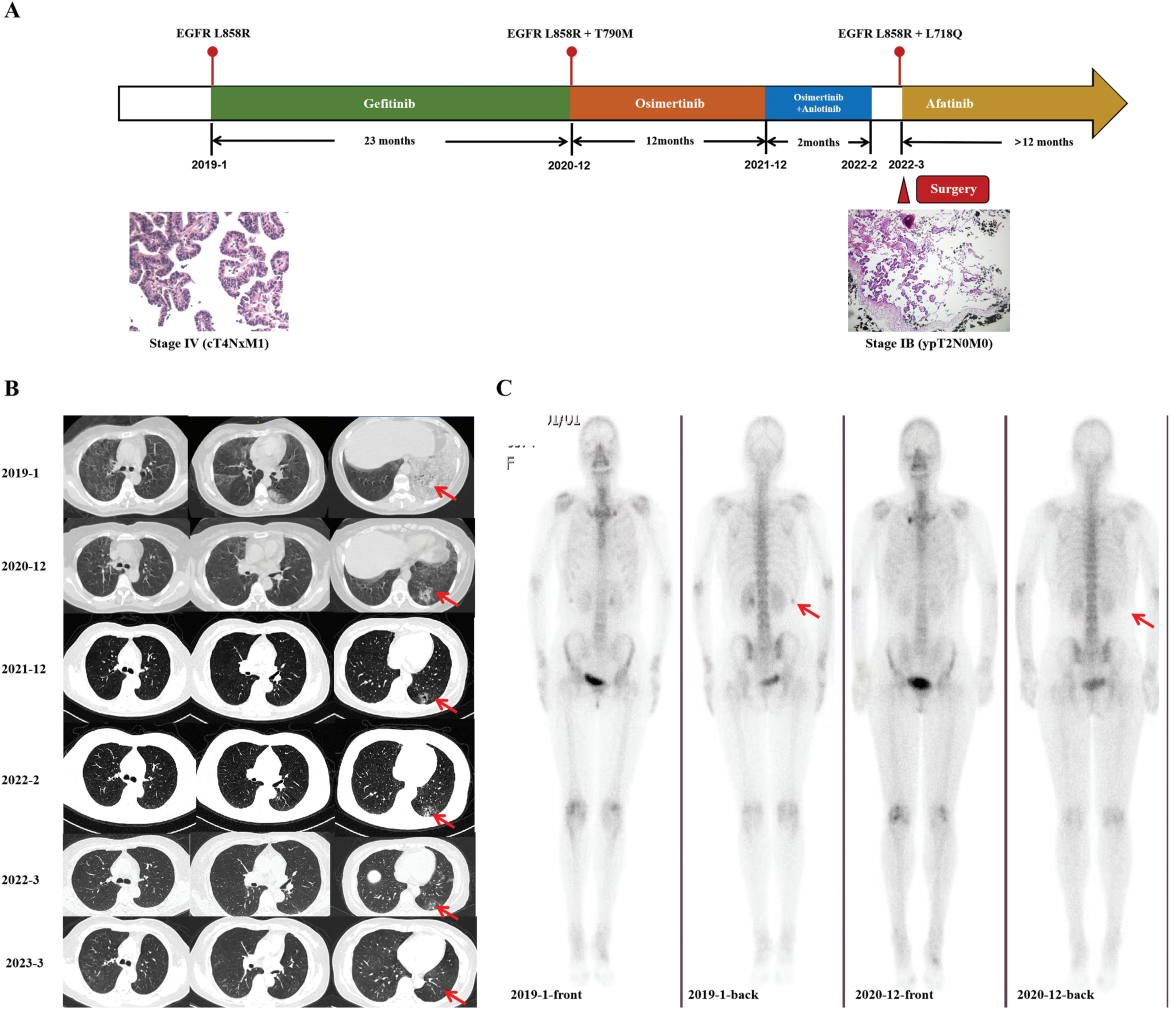
Evolution of the disease in case presentation. (**A**) Treatment course and corresponding clinical features of the patient after EGFR mutation was detected. (**B**) Computed tomography (CT) imaging of the course of disease. (**C**) Bone scan features of the patient before and after gefitinib.

A 55-year-old Chinese female patient, a nonsmoker teacher, was diagnosed with stage IV (cT4NxM1) lung adenocarcinoma, which presented with diffuse bilateral lung lesions and a right 11th rib metastasis, at our hospital in January 2019. The likely primary site was the left lower lobe. The patient was given gefitinib at 250 mg/qd as a first-line treatment after identification of the epidermal growth factor receptor (EGFR) L858R mutation in a tumor biopsy sample using the amplification-refractory mutation system (Fig. 1A). Lesions in the left lower lobe were gradually reduced in size and a cavity appeared in the center. This was considered a partial response (PR) (Fig. 1B). Re-examination in December 2020 revealed an enlarging lesion in the left lower lobe, and the patient was assessed as having disease progression. The original radioactive focus in the right 11th rib had disappeared on bone scan (Fig. 1C). Computed tomography (CT)-guided left lower lung biopsy was reperformed, and next-generation sequencing (NGS) revealed L858R (mutant allele frequency [MAF]: 49.20%) and T790M (MAF: 21.60%) mutations. Hence, treatment was switched to osimertinib at 80 mg/qd, which achieved PR. Unfortunately, the patient’s disease progressed again after 12 months of osimertinib treatment in December 2021. Anlotinib was added to osimertinib and repeat CT imaging revealed a solidified and enlarged left lower lung cavity lesion. However, systemic examination still revealed no evidence of distant metastasis. The patient underwent a thoracoscopic lobectomy and systematic lymph node dissection in March 2022. The postoperative pathology was adenocarcinoma, ~1.9 cm × 1.8 cm × 1 cm in size with pleural invasion. Fortunately, the staples, broken ends of the vessels, and broken ends of the bronchus revealed no tumor, with no tumor thrombus or nerve invasion. The 5th, 7th, 9th, 10th, and 11th lymph node groups demonstrated no metastasis. The postoperative diagnosis was ypT2N0M0 stage IB. NGS testing on surgical tissue revealed that the EGFR L858R (MAF: 31.37%) mutation remained, the T790M mutation had vanished, and a new EGFR L718Q (MAF: 27.5%) mutation had emerged. Previous reports suggested that L718Q conferred osimertinib resistance, but may still be sensitive to afatinib.^1,2^ Thus, the patient was prescribed afatinib at 40 mg/qd as postoperative treatment, and no tumor recurrence or metastasis was found through March 2023.

## Discussion

This case report shows the value of gene mutation testing during EGFR-tyrosine kinase inhibitor (EGFR-TKI) sequential treatment. The treatment was switched to osimertinib after gefitinib resistance emerged. The latest version of the National Comprehensive Cancer Network guidelines in 2023 recommends osimertinib as the first choice for systemic treatment in patients with non–small cell lung cancer (NSCLC) harboring an EGFR L858R mutation. Hence, patients will be starting treatment with osimertinib. Our patient acquired an EGFR L718Q mutation, which resulted in subsequent osimertinib resistance. The EGFR L718Q mutation confers resistance to osimertinib by altering the EGFR-osimertinib complex conformation, preventing the reaction with C797. Several studies have revealed that tumors bearing L718Q mutations may be sensitive to afatinib and dacomitinib.^1-3^ Therefore, afatinib was prescribed postoperatively for our patient. In all, our patient has benefited from EGFR-TKIs for >51 months from the time of diagnosis to the present.

Oligometastasis is a concept proposed by Hellman and Weichselbaum^4^, which refers to a transitional state between localized and extensive metastases. The brain, bone, adrenal gland, liver, and other rare sites are the common extrapulmonary metastasis organs of NSCLC. Oligometastatic NSCLC differs from the traditional advanced state of lung cancer as a potentially curable disease, highlighting the role of local consolidation therapy such as surgery or radiotherapy.^5-7^

Clinical studies have revealed improved survival with the addition of local curative therapy to standard systemic therapy in oligometastatic NSCLC.^8,9^ Gomez et al^6,7^ revealed that combining local consolidative therapy with radiation or surgery significantly prolonged progression-free survival (PFS) and overall survival (OS) compared with maintenance therapy/observation in patients with oligometastatic NSCLC who did not progress after first-line systemic therapy such as EGFR-TKI. The CLAT study revealed consolidative local ablative therapy (LAT) to all metastatic sites as a feasible option for patients with EGFR-mutant oligometastatic NSCLC during first-line EGFR-TKI treatment, with significantly improved PFS and OS compared with the consolidative LAT to partial sites or observation alone.^10^ Yu et al^11^ reported that EGFR-mutant lung cancers with acquired resistance to EGFR-TKI therapy are amenable to local therapy to treat oligometastatic disease when combined with continued EGFR inhibition. Local therapy followed by continued EGFR-TKI treatment is well-tolerated and associated with long PFS and OS. In conclusion, patients with oligometastasis who are treated with targeted therapy, such as EGFR-TKI and ALK-TKI, in conjunction with local therapy, may have better efficacy.^12^ However, reports of tumor downstaging resulting from the elimination of metastases by EGFR-TKI are uncommon. We chose surgical resection when our patient developed progression in 2022 because of the benefit of EGFR-TKI and the limited lesion in the left lower lobe. The patient recovered well postoperatively, and postoperative pathology revealed stage IB. Therefore, our case suggests that sequential targeted therapy is safe and feasible for achieving downstaging and surgical outcomes in EGFR-mutated NSCLC with oligometastatic disease.

## Data Availability

The data underlying this article will be shared on reasonable request to the corresponding author.

## References

[1] Liu J , Jin B, Su H, Qu X, Liu Y. Afatinib helped overcome subsequent resistance to osimertinib in a patient with NSCLC having leptomeningeal metastasis baring acquired EGFR L718Q mutation: a case report. BMC Cancer. 2019;19(1):702. 10.1186/s12885-019-5915-7 31315676 PMC6637526

[2] Yang X , Huang C, Chen R, Zhao J. Resolving resistance to osimertinib therapy with afatinib in an NSCLC patient with EGFR L718Q mutation. Clin Lung Cancer. 2020;21(4):e258-e260. 10.1016/j.cllc.2019.12.002 32146032

[3] Shen Q , Qu J, Chen Z, Zhou J. Case Report: dacomitinib overcomes osimertinib resistance in NSCLC patient harboring L718Q mutation: a case report. Front Oncol. 2021;11:760097. 10.3389/fonc.2021.760097 34926262 PMC8674200

[4] Hellman S , Weichselbaum RR. Oligometastases. J Clin Oncol. 1995;13(1):8-10. 10.1200/JCO.1995.13.1.8 7799047

[5] Wong AC , Watson SP, Pitroda SP, et al. Clinical and molecular markers of long-term survival after oligometastasis-directed stereotactic body radiotherapy (SBRT). Cancer. 2016;122(14):2242-2250. 10.1002/cncr.30058 27206146

[6] Gomez DR , Blumenschein GR Jr, Lee JJ, et al. Local consolidative therapy versus maintenance therapy or observation for patients with oligometastatic non-small-cell lung cancer without progression after first-line systemic therapy: a multicentre, randomised, controlled, phase 2 study. Lancet Oncol. 2016;17(12):1672-1682. 10.1016/S1470-2045(16)30532-0 27789196 PMC5143183

[7] Gomez DR , Tang C, Zhang J, et al. Local consolidative therapy vs maintenance therapy or observation for patients with oligometastatic non-small-cell lung cancer: long-term results of a multi-institutional, phase II, randomized study. J Clin Oncol. 2019;37(18):1558-1565. 10.1200/JCO.19.00201 31067138 PMC6599408

[8] Lievens Y , Guckenberger M, Gomez D, et al. Defining oligometastatic disease from a radiation oncology perspective: an ESTRO-ASTRO consensus document. Radiother Oncol. 2020;148:157-166. 10.1016/j.radonc.2020.04.003 32388150

[9] Guckenberger M , Lievens Y, Bouma AB, et al. Characterisation and classification of oligometastatic disease: a European Society for Radiotherapy and Oncology and European Organisation for Research and Treatment of Cancer consensus recommendation. Lancet Oncol. 2020;21(1):e18-e28. 10.1016/S1470-2045(19)30718-1 31908301

[10] Xu Q , Zhou F, Liu H, et al. Consolidative local ablative therapy improves the survival of patients with synchronous oligometastatic NSCLC harboring EGFR activating mutation treated with first-line EGFR-TKIs. J Thorac Oncol. 2018;13(9):1383-1392. 10.1016/j.jtho.2018.05.019 29852232

[11] Yu HA , Sima CS, Huang J, et al. Local therapy with continued EGFR tyrosine kinase inhibitor therapy as a treatment strategy in EGFR-mutant advanced lung cancers that have developed acquired resistance to EGFR tyrosine kinase inhibitors. J Thorac Oncol. 2013;8(3):346-351. 10.1097/JTO.0b013e31827e1f83 23407558 PMC3673295

[12] Suzuki H , Yoshino I. Approach for oligometastasis in non-small cell lung cancer. Gen Thorac Cardiovasc Surg. 2016;64(4):192-196. 10.1007/s11748-016-0630-7 26895202

